# Supplementary material for: The immune checkpoint B7x expands tumor-infiltrating Tregs and promotes resistance to anti-CTLA-4 therapy
Source: Nat Commun. 2022 May 6;13:2506. doi: 10.1038/s41467-022-30143-8 (PMC9076640; doi:10.1038/s41467-022-30143-8)
Supplement: Supplementary file 1 — Supplementary Information [file 41467_2022_30143_MOESM1_ESM.pdf]

Supplementary Information for

The immune checkpoint B7x expands tumor-infiltrating Tregs and promotes resistance to anti-CTLA-4 therapy

Peter John, Marc C. Pulanco, Phillip M. Galbo Jr., Yao Wei, Kim C. Ohaegbulam, Deyou Zheng, Xingxing Zang

Corresponding author: Xingxing Zang, xingxing.zang@einsteinmed.edu

This PDF file includes:

Supplementary Figures 1-6
Supplementary Table 1

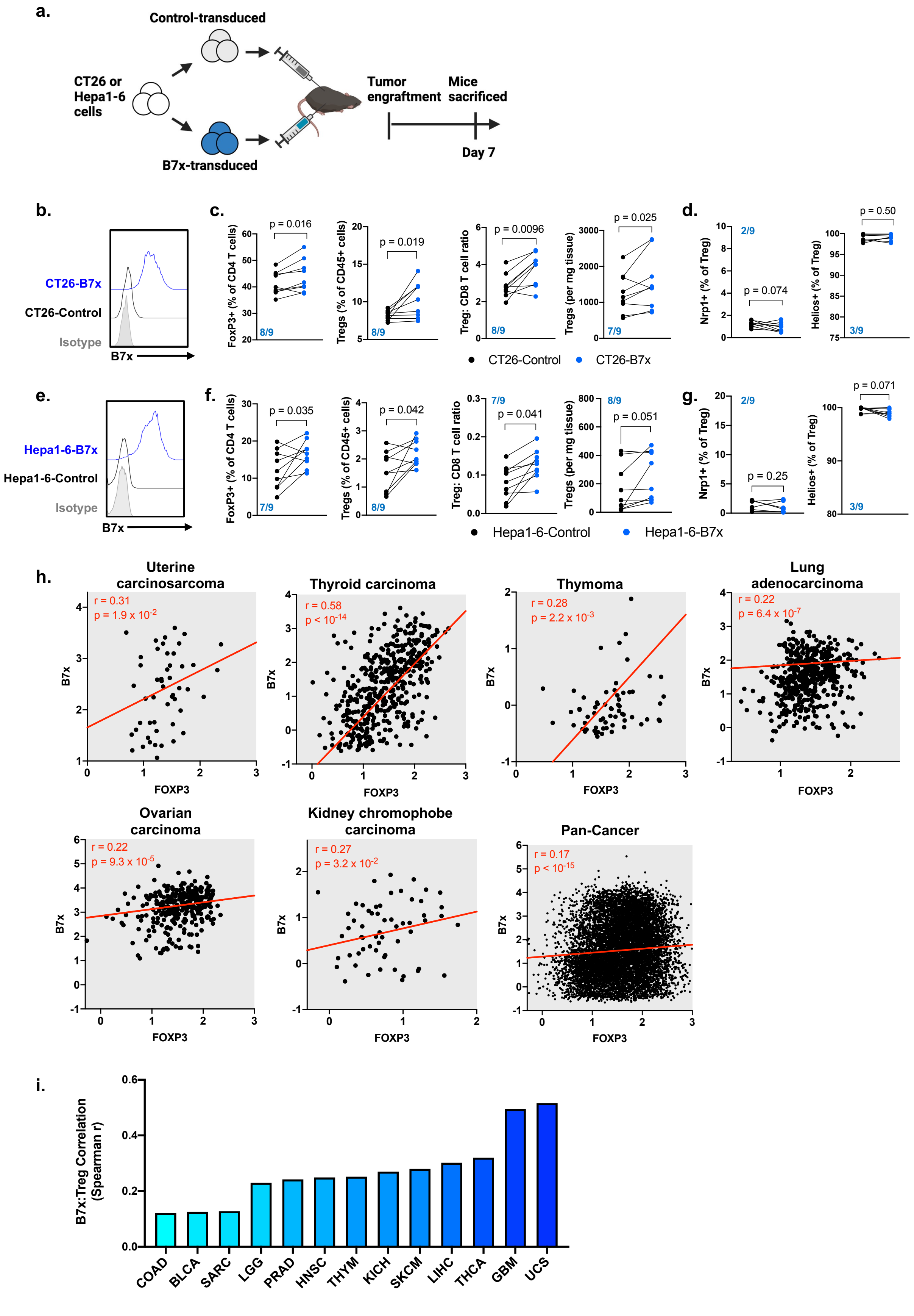

a.
Control-transduced
CT26 or Hepa1-6 cells
B7x-transduced
Tumor engraftment
Mice sacrificed
Day 7
b.
CT26-B7x
CT26-Control
Isotype
B7x
c.
p = 0.016
p = 0.019
p = 0.0096
p = 0.025
FoxP3+ (% of CD4 T cells)
Tregs (% of CD45+ cells)
Treg: CD8 T cell ratio
Tregs (per mg tissue)
8/9
8/9
8/9
7/9
CT26-Control
CT26-B7x
d.
p = 0.074
p = 0.50
Nrp1+ (% of Treg)
Helios+ (% of Treg)
2/9
3/9
e.
Hepa1-6-B7x
Hepa1-6-Control
Isotype
B7x
f.
p = 0.035
p = 0.042
p = 0.041
p = 0.051
7/9
8/9
7/9
8/9
Hepa1-6-Control
Hepa1-6-B7x
g.
p = 0.25
p = 0.071
2/9
3/9
h.
Uterine carcinosarcoma
r = 0.31
p = 1.9 x 10-2
Thyroid carcinoma
r = 0.58
p < 10-14
Thymoma
r = 0.28
p = 2.2 x 10-3
Lung adenocarcinoma
r = 0.22
p = 6.4 x 10-7
Ovarian carcinoma
r = 0.22
p = 9.3 x 10-5
Kidney chromophobe carcinoma
r = 0.27
p = 3.2 x 10-2
Pan-Cancer
r = 0.17
p < 10-15
B7x
FOXP3
i.
B7x:Treg Correlation (Spearman r)
COAD
BLCA
SARC
LGG
PRAD
HNSC
THYM
KICH
SKCM
LIHC
THCA
GBM
UCS

Supplementary Figure 1. Tumor-expressed B7x promotes infiltrating Treg populations

a. Experimental scheme of CT26 and Hepa1-6 engraftment experiments. **b.** Representative flow cytometric analysis of B7x expression in CT26-B7x and CT26-Control cells. **c, d.** Tumor-infiltrating T cell populations and functional markers in CT26 tumors were analyzed (*n* = 9 per group), fraction of mice for which the B7x tumors show an increase in the respective measurement relative to the Control tumors are displayed in corners of graphs. **e.** Representative flow cytometric analysis of B7x expression in Hepa1-6-B7x and Hepa1-6-Control cells **f, g.** Tumor-infiltrating T cell populations and functional markers in Hepa1-6 tumors were analyzed as described in **c, d** (*n* = 9 per group)**.** P values for bar graphs were calculated by ratio paired parametric T-test. **h.** RNA-seq expression data for Foxp3 and B7x from the TCGA database was analyzed for uterine carcinosarcoma (*n* = 57), thyroid carcinoma (*n* = 498), thymoma (n = 119), lung adenocarcinoma (*n* = 493), ovarian carcinoma (n = 303), kidney chromophobe carcinoma (n = 65), and a pan-cancer analysis of 30 cancers (*n* = 9812). Best-fit log lines are displayed in red. Spearman's r and two-tailed P values are designated in the upper-left corner. **i.** Correlation of B7x with quanTIseq Treg gene signature in TCGA data sets, ranked by Spearman's r. TCGA data set abbreviations are defined in the Methods section.

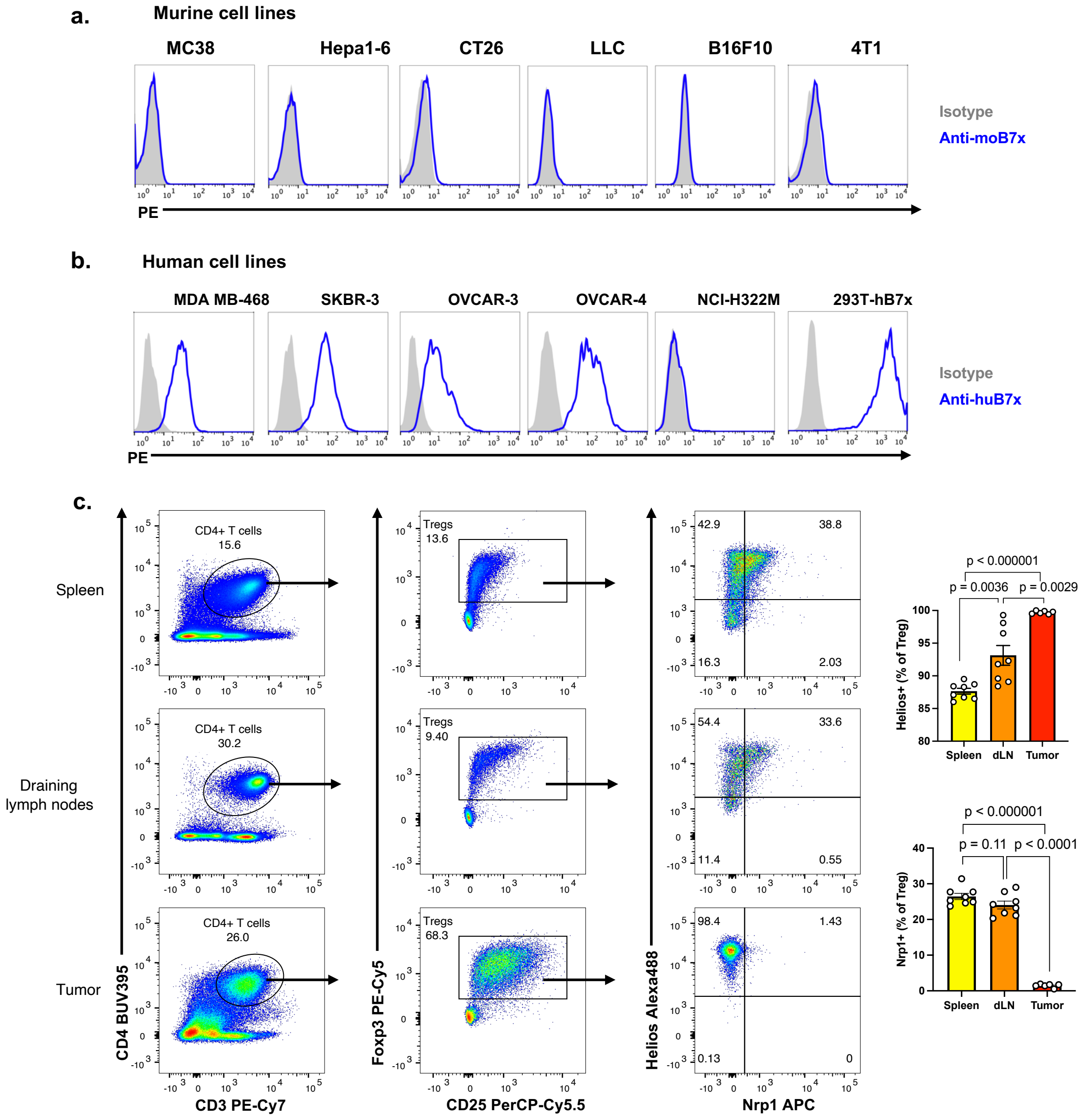

Supplementary Figure 2. Expression of B7x in cancer cell lines and lineage markers Nrp1 and Helios in Tregs

a. Expression of B7x in murine tumor cell lines commonly used in syngeneic models was analyzed by flow cytometry. **b.** Expression of B7x in B7x^{+} human tumor cell lines, including B7x^{-} negative control (NCI-H322M) and stably transduced positive control (293T-hB7x). **c.** Mice were engrafted with MC38 tumors, were sacrificed after 7 days, and Treg populations in spleens, draining lymph nodes, and tumors were analyzed by flow cytometry. *n* = 7 mice, each group represents a separate organ. Error bars represent SEM, P values were calculated by Student's T-test.

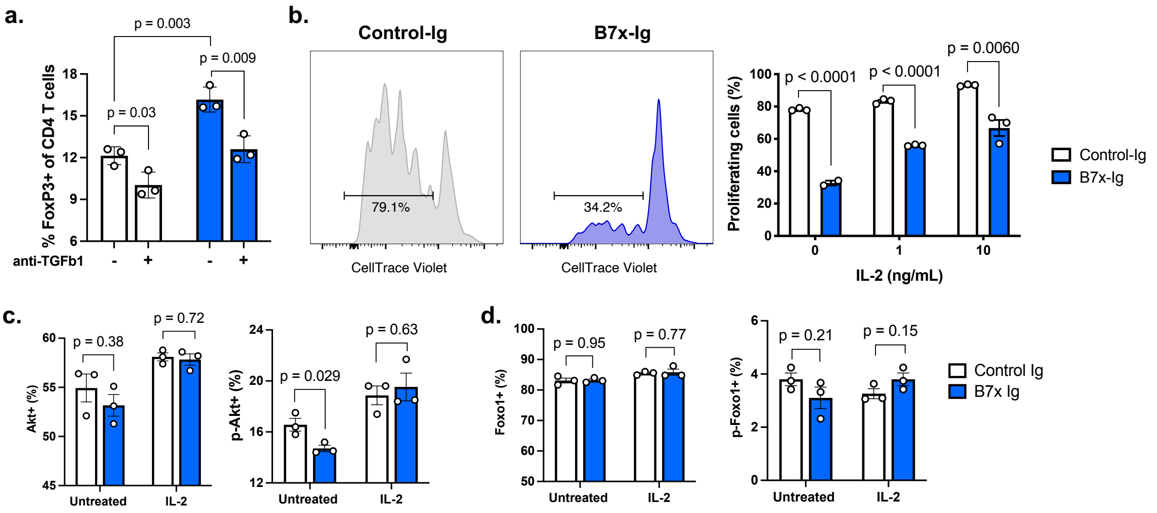

Supplementary Figure 3. B7x inhibits nTreg proliferation and activation

a. $CD4^+$ T cells were cultured with anti-CD3, anti-CD28, and either B7x-transduced or control MC38 or Hepa1-6 cells, and either anti-TGFb1 (+) or IgG isotype (-) antibody. Expression of Foxp3 in the T cells was analyzed after 4 days. **b-d.** Splenic nTregs (GFP^+ $CD4^+$T cells) were isolated from Foxp3-GFP/DTR mice, were stained with CTV, stimulated to proliferate for 3 days with anti-CD3/CD28 Dynabeads, after which proliferation was measured by dye dilution (**b**). After 24hr, nTregs were analyzed by phospho-flow cytometry for Akt and p-Akt (**c**) or Foxo1 and p-Foxo1 (**d**). Each point in **a-d** represent technical replicates from the representative experiments, performed in triplicates (n = 3 per group). Error bars represent SEM, P values were calculated by two-tailed Student's T-test.

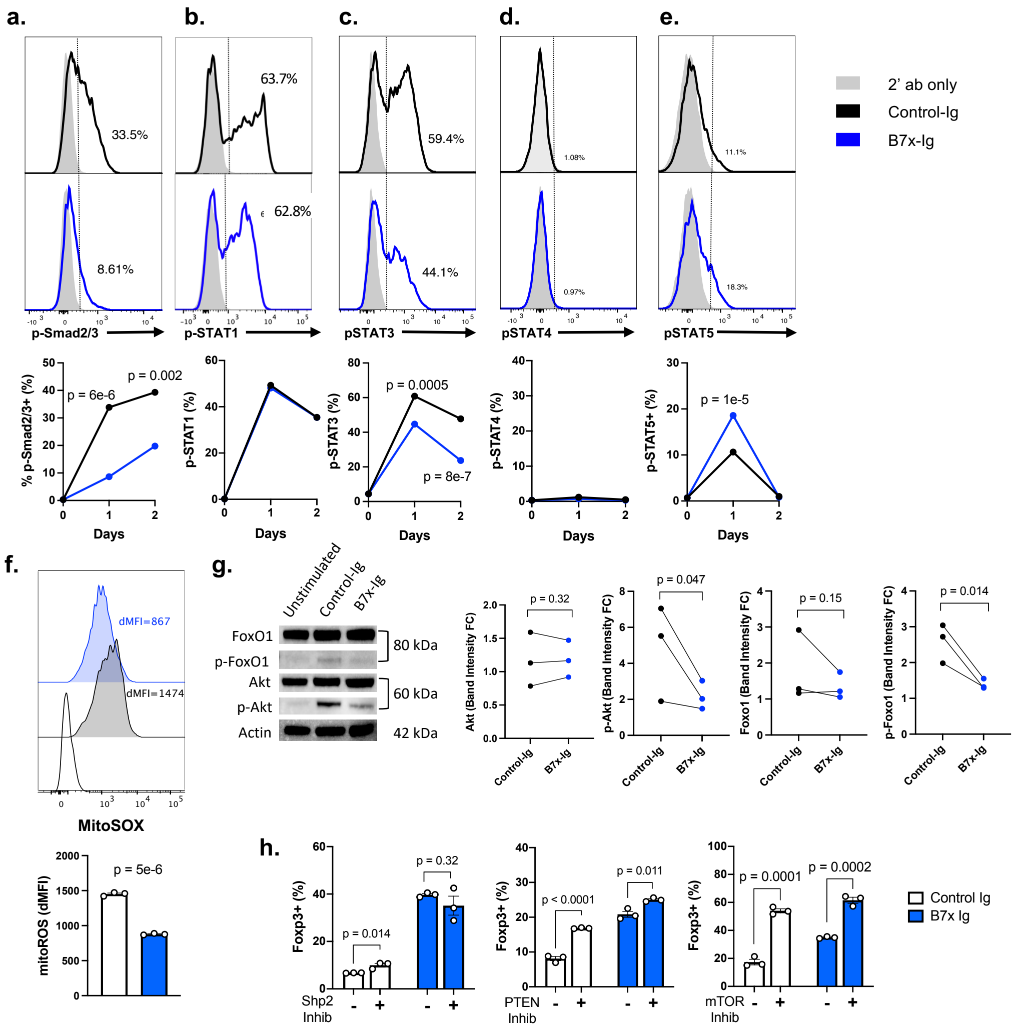

Supplementary Figure 4. B7x modulates phosphorylation of cytokine-pathway transcription factors

a-e. CD4$^+$ T cells were cultured in iTreg-inducing conditions with either B7x-Ig or Control-Ig for up to 48 hours, and phosphorylation status of Smad2/3 (**a**), STAT1 (**b**), STAT3 (**c**), STAT4 (**d**), and STAT5 (**e**). **f.** CD4$^+$ T cells were cultured in iTreg-inducing conditions as described in **a,** were stained with mitoSOX after 48 hours, and staining intensity was measured by flow cytometry (top); delta mean fluorescence intensity (MFI stained – MFI unstained) was calculated (bottom). **g.** CD4$^+$ T cells were cultured in iTreg-inducing conditions for 24 hours with either Control-Ig or B7x-Ig, after which cells were lysed and analyzed by western blot and phospho-blotting. Representative blots are shown (left), and band intensity was measured in three independent experiments (right). **h.** CD4$^+$ T cells were cultured in iTreg-inducing conditions with either B7x-Ig or Control-Ig and were treated with inhibitors (+) against Shp2 (Shp099), PTEN (SF1670), and mTOR (rapamycin) or vehicle control (-). Each point in (**h**) represent technical replicates from the representative experiments, performed in triplicates (n = 3 per group). Error bars represent SEM, P values were calculated by two-tailed Student's T-test.

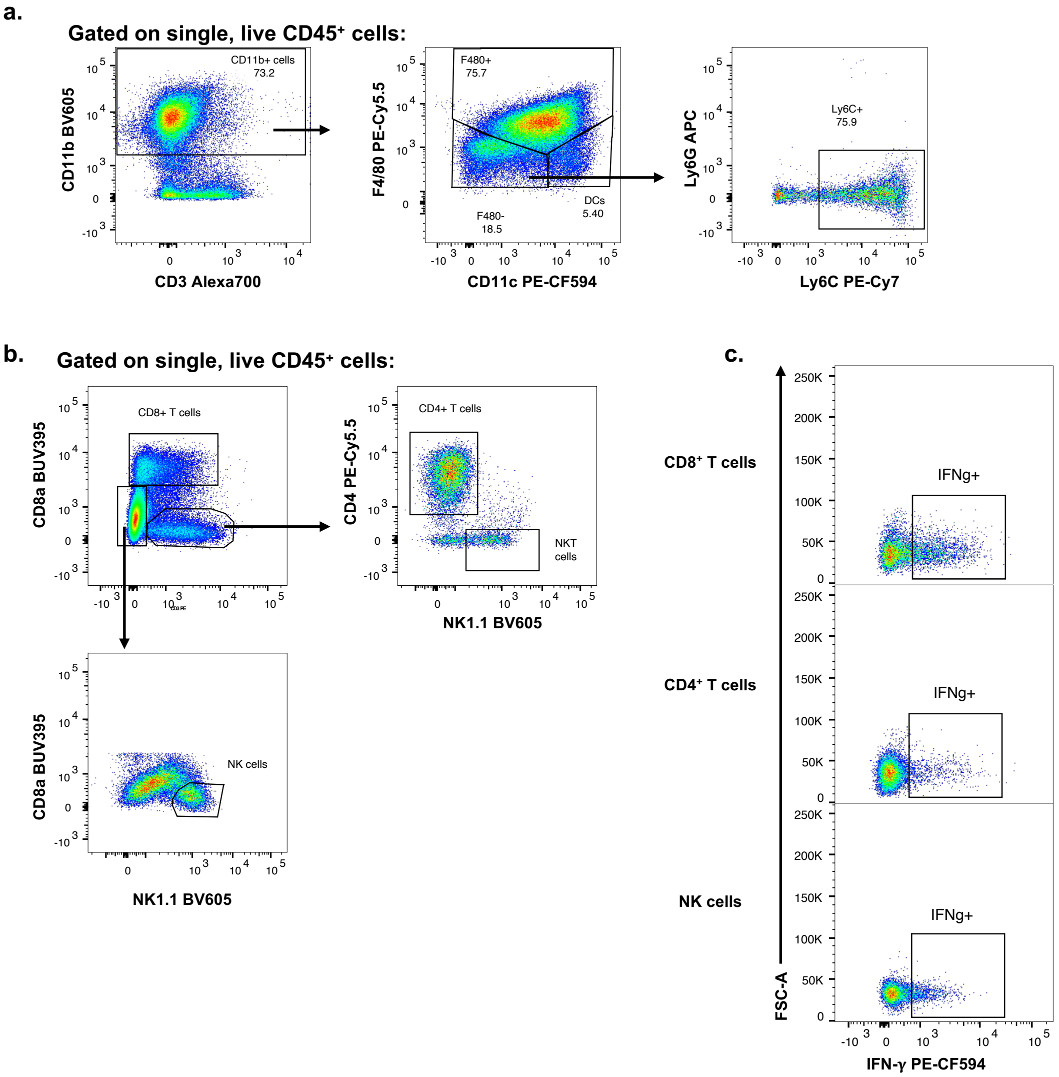

Supplementary Figure 5. Gating strategies for tumor-infiltrating immune cells

a-c. Representative gating strategies for $CD45^{+}$ immune cell populations in dissociated MC38 tumors. **a.** Myeloid cell gating strategy, for macrophages, dendritic cells, and monocytes. **b.** Lymphocyte gating strategy for $CD8^{+}$ T cells, $CD4^{+}$ T cells, NK cells, and NK T cells. **c.** Cell suspensions were stimulated with PMA ionomycin, and expression of IFN-γ was analyzed in effector immune cell subsets.

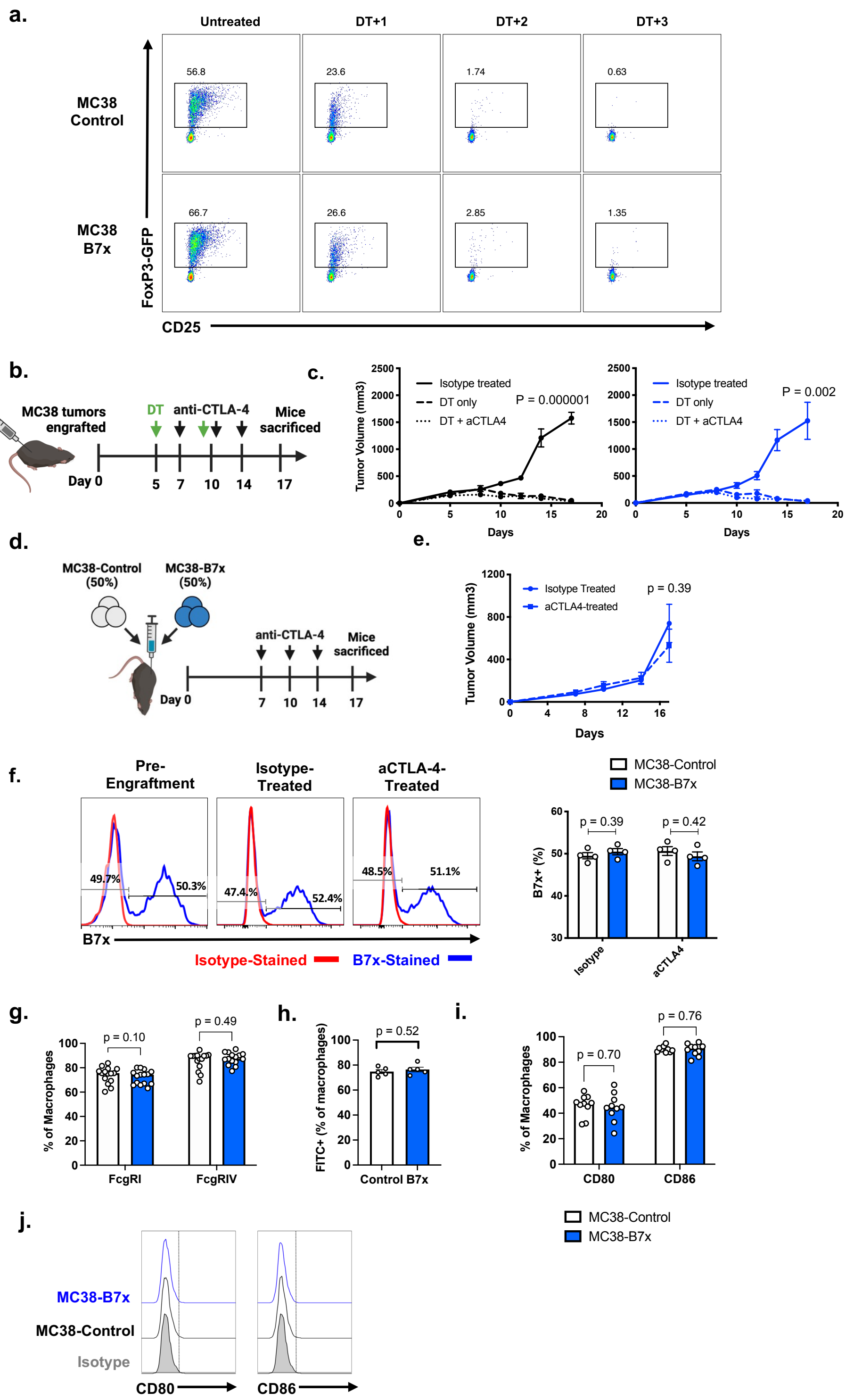

a.
Untreated
DT+1
DT+2
DT+3
MC38 Control
MC38 B7x
FoxP3-GFP
CD25
56.8
23.6
1.74
0.63
66.7
26.6
2.85
1.35
b.
MC38 tumors engrafted
DT
anti-CTLA-4
Mice sacrificed
Day 0
5
7
10
14
17
c.
Tumor Volume (mm3)
Isotype treated
DT only
DT + aCTLA4
P = 0.000001
P = 0.002
Days
d.
MC38-Control (50%)
MC38-B7x (50%)
anti-CTLA-4
Mice sacrificed
Day 0
e.
Isotype Treated
aCTLA4-treated
p = 0.39
Tumor Volume (mm3)
Days
f.
Pre-Engraftment
Isotype-Treated
aCTLA-4-Treated
49.7%
50.3%
47.4.%
52.4%
48.5%
51.1%
B7x
Isotype-Stained
B7x-Stained
MC38-Control
MC38-B7x
p = 0.39
p = 0.42
B7x+ (%)
Isotype
aCTLA4
g.
p = 0.10
p = 0.49
% of Macrophages
FcgRI
FcgRIV
h.
p = 0.52
FITC+ (% of macrophages)
Control B7x
i.
p = 0.70
p = 0.76
% of Macrophages
CD80
CD86
j.
MC38-B7x
MC38-Control
Isotype
CD80
CD86
MC38-Control
MC38-B7x

Supplementary Figure 6. B7x-mediated resistance to anti-CTLA-4 therapy requires Tregs

a. Foxp3-GFP/DTR mice were engrafted with MC38 tumors and were treated with 20 ng/g diptheria toxin (DT) to deplete Foxp3$^+$ Tregs. Populations of GFP$^+$ Tregs was tracked after 1, 2, and 3 days following DT treatment. **b.** Foxp3-GFP/DTR mice were engrafted with MC38 tumors as in (**a**), and were treated with anti-CTLA-4 or IgG isotype antibody, **c.** and tumor volumes were tracked. *n* = 4 in isotype-treated group, 6 in DT and DT+ anti-CTLA-4-treated groups. **d.** MC38-B7x and MC38-Control tumor cells were mixed 1:1 and engrafted into wild type mice, which were subsequently treated with anti-CTLA-4 or IgG isotype antibody. **e.** Tumor volumes were tracked (*n* = 8 per group). **f.** Mice were engrafted with tumors and treated as in **(d)**, after which tumors were dissociated, and fractions of B7x$^+$ and B7x$^-$ tumor cells were quantified (*n* = 4 tumors per group). **g.** Mice were engrafted with MC38-B7x or MC38-Control tumors, and tumors were dissociated after 7 days. Expression of Fc receptors were analyzed in F4/80$^+$ tumor-infiltrating macrophages. *n* = 12 mice per group. **h.** CD11b$^+$ F4/80$^+$ macrophages were flow-sorted and co-cultured with FITC-labeled pHrodo phagocytosis beads, and % of FITC$^+$ macrophages were analyzed after 2 hours. Each point represents a biological replicate using macrophages extracted from distinct tumor-bearing mice, *n* = 5 mice per group. **i.** Mice were engrafted with MC38-Control or MC38-B7x tumors, after which tumors were dissociated and F4/80$^+$ macrophages were analyzed for expression of CD80 and CD86. *n* = 10 per group, representative of 2 independent experiments. **j.** MC38-Control and MC38-B7x tumor cell lines were analyzed for expression of CD80 and CD86. Error bars represent SEM, P values were calculated by two-tailed Student's T-test.

Supplementary Table 1. Antibodies used for flow cytometry

Table 1a. Antibodies used for surface staining

Target species	Target protein	Clone	Fluorophore	Dilution	Manufacturer	Catalog Number
Mouse	B220	RA3-6B2	PerCP-Cy5.5	1:400	Biolegend	103235
Mouse	B7x	HMH4-5G1	PE	1:200	Biolegend	358103
Mouse	CD11b	M1/70	PE	1:400	Biolegend	101207
Mouse	CD11c	N418	FITC	1:400	Biolegend	117305
Mouse	CD206	C068C2	PE	1:200	Biolegend	141705
Mouse	CD25	PC61	PerCP-Cy5.5	1:200	Biolegend	101911
Mouse	CD3	17A2	PE-Cy7	1:400	Biolegend	300419
Mouse	CD4	GK1.5	APC,	1:400	Biolegend	100411
Mouse	CD45	30-F11	PE, V450	1:400	BD	560501
Mouse	CD45.1	A20	Alexa700	1:400	Biolegend	110723
Mouse	CD45.2	104	PE	1:400	Biolegend	109807
Mouse	CD8a	53-6.7	BUV395	1:400	BD	565968
Mouse	F4/80	BM8	PE-Cy5.5	1:400	Biolegend	123111
Mouse/human	Foxo1*	C29H4	Unconjugated	1:100	CST	2880T
Mouse	Ly6C	HK1.4	PE-Cy7	1:800	Biolegend	128017
Mouse	Ly6G	1A8	APC	1:400	Biolegend	108411
Mouse	Neuropilin1	3E12	APC	1:200	Biolegend	145205
Mouse	NK1.1	PK136	Alexa700	1:200	Biolegend	108729
Mouse	PD-L1	10F.9G2	PE, BV421	1:200	Biolegend	124307
Mouse	PD-1	29F.1A12	PE	1:200	Biolegend	135205
Mouse	TGF-LAP	TW7-20B9	PE	1:200	Biolegend	141403
Mouse	Tim3	B8.2C12	APC	1:200	Tonbo	20-5870
Human	B7x	MIH43	PE	1:200	Biolegend	358103
Human	PD-L1	MIH1	PE	1:200	Biolegend	329706
Rabbit	IgG (2' ab)	Polyclonal	Alexa647	1:200	Thermofisher	A-21237

*Used with phospho-flow protocol. Also used for microscopy.

Table 1b. Antibodies used for intracellular staining

Target species	Target protein	Clone	Fluorophore	Dilution	Manufacturer	Catalog Number
Mouse	CD107a	1D4B	PerCP-Cy5.5	1:100	Biolegend	121625
Mouse	Foxp3	FJK-16s	PE-Cy5.5	1:100	Thermofisher	35-5773-82
Mouse/human	Helios	22F6	Alexa488	1:200	BD	563950
Mouse	IFN-γ	XMG1.2	PE-CF594	1:100	BD	562333
Mouse	Ki67	16A8	BV605	1:200	Biolegend	652413

Table 1c. Antibodies used for phospho-flow cytometry

Target species	Target phospho-protein	Clone	Dilution	Manufacturer	Catalog Number
Mouse/human	Akt (Ser473)	9271	1:100	CST	9271T
Mouse/human	c-Jun (Ser73)	D47G9	1:100	CST	3270T
Mouse/human	Foxo1 (Ser256)	9461	1:100	CST	9461T
Mouse/human	p65 (Ser536)	93H1	1:100	CST	3033T
Mouse/human	STAT1 (Tyr3701)	58D6	1:100	CST	9167S
Mouse/human	STAT3 (Tyr705)	4/P-STAT3	1:100	BD	557815
Mouse/human	STAT4 (Tyr693)	5267	1:100	CST	4134S
Mouse/human	STAT5 (Tyr694)	C11C5	1:100	CST	9359S
Mouse/human	Smad2/3 (Ser465/423)	O72-670	1:100	BD	562696
