# Supplementary material for: The immune checkpoint B7x expands tumor-infiltrating Tregs and promotes resistance to anti-CTLA-4 therapy
Source: Nat Commun. 2022 May 6;13:2506. doi: 10.1038/s41467-022-30143-8 (PMC9076640; doi:10.1038/s41467-022-30143-8)
Supplement: Supplementary file 2 — Reporting Summary [file 41467_2022_30143_MOESM2_ESM.pdf]

nature portfolio

Corresponding author(s): Xingxing Zang

Last updated by author(s): Mar 30, 2022

Reporting Summary

Nature Portfolio wishes to improve the reproducibility of the work that we publish. This form provides structure for consistency and transparency in reporting. For further information on Nature Portfolio policies, see our Editorial Policies and the Editorial Policy Checklist.

Statistics

For all statistical analyses, confirm that the following items are present in the figure legend, table legend, main text, or Methods section.

n/a	Confirmed	
[ ]	[x]	The exact sample size (*n*) for each experimental group/condition, given as a discrete number and unit of measurement
[ ]	[x]	A statement on whether measurements were taken from distinct samples or whether the same sample was measured repeatedly
[ ]	[x]	The statistical test(s) used AND whether they are one- or two-sided<br>*Only common tests should be described solely by name; describe more complex techniques in the Methods section.*
[ ]	[x]	A description of all covariates tested
[ ]	[x]	A description of any assumptions or corrections, such as tests of normality and adjustment for multiple comparisons
[ ]	[x]	A full description of the statistical parameters including central tendency (e.g. means) or other basic estimates (e.g. regression coefficient) AND variation (e.g. standard deviation) or associated estimates of uncertainty (e.g. confidence intervals)
[ ]	[x]	For null hypothesis testing, the test statistic (e.g. *F*, *t*, *r*) with confidence intervals, effect sizes, degrees of freedom and *P* value noted<br>*Give P values as exact values whenever suitable.*
[x]	[ ]	For Bayesian analysis, information on the choice of priors and Markov chain Monte Carlo settings
[x]	[ ]	For hierarchical and complex designs, identification of the appropriate level for tests and full reporting of outcomes
[ ]	[x]	Estimates of effect sizes (e.g. Cohen's *d*, Pearson's *r*), indicating how they were calculated

Our web collection on statistics for biologists contains articles on many of the points above.

Software and code

Policy information about availability of computer code

Data collection	BD FACS Diva 8.0 for flow cytometry acquisition, Leica LAS X for microscopy imaging acquisition
Data analysis	Flowjo 10.7.0 for flow cytometry analysis, Graphpad Prism 8.4.3 for statistical analysis and data visualization, FIJI (ImageJ) V2.0.0 for microscopy analysis, GSEA 4.1.0 for GSEA analysis, STAR for RNA-seq alignment, HTseq and DESeq2v3.11 for RNA-seq analysis, cBioPortal for TCGA data sets, TCGA gene signature correlation by quanTIseq

For manuscripts utilizing custom algorithms or software that are central to the research but not yet described in published literature, software must be made available to editors and reviewers. We strongly encourage code deposition in a community repository (e.g. GitHub). See the Nature Portfolio guidelines for submitting code & software for further information.

Data

Policy information about availability of data

All manuscripts must include a data availability statement. This statement should provide the following information, where applicable:

- Accession codes, unique identifiers, or web links for publicly available datasets
- A description of any restrictions on data availability
- For clinical datasets or third party data, please ensure that the statement adheres to our policy

The sequencing data that support findings in this study are deposited in the Gene Expression Omnibus (GSE199751). Source data for all graphs are provided as Source Data files. All other data supporting the findings of this study are available from the corresponding author upon request.

Field-specific reporting

Please select the one below that is the best fit for your research. If you are not sure, read the appropriate sections before making your selection.

☒ Life sciences ☐ Behavioural & social sciences ☐ Ecological, evolutionary & environmental sciences

For a reference copy of the document with all sections, see nature.com/documents/nr-reporting-summary-flat.pdf

Life sciences study design

All studies must disclose on these points even when the disclosure is negative.

Sample size	Sample sizes were determined based on the results of preliminary experiments, no statistical method was used to determine sample size
Data exclusions	One animal was excluded from Fig. 1a, 1h due to being statistical outliers as determined by Grubb's outlier test. For flow cytometric analyses, samples with very sparse cell populations, very poor cell viability, or poor quality sample preparation were excluded. All data exclusion criteria were pre-determined.
Replication	In vivo experiments were repeated at least once after preliminary experiments with consistent data. In vitro experiments were successfully repeated three or more times, representative data are shown.
Randomization	For all tumor experiments, age-matched and sex-matched animals were randomly distributed into Control or B7x groups on the day of tumor engraftment. Animals used for experimental therapeutics experiments were randomized into control or treated groups 7 days after tumor engraftment, prior to the start of treatment.
Blinding	Investigators were not blinded to group allocation during data collection or analysis, as investigators had to be aware of the groups during separation and treatment steps. Blinding was not needed for data analysis since identical or comparable analysis criteria were applied to all groups.

Reporting for specific materials, systems and methods

We require information from authors about some types of materials, experimental systems and methods used in many studies. Here, indicate whether each material, system or method listed is relevant to your study. If you are not sure if a list item applies to your research, read the appropriate section before selecting a response.

Materials & experimental systems

n/a	Involved in the study	
☐	☒	Antibodies
☐	☒	Eukaryotic cell lines
☒	☐	Palaeontology and archaeology
☐	☒	Animals and other organisms
☒	☐	Human research participants
☒	☐	Clinical data
☒	☐	Dual use research of concern

Methods

n/a	Involved in the study	
☒	☐	ChIP-seq
☐	☒	Flow cytometry
☒	☐	MRI-based neuroimaging

Antibodies

Antibodies used	Functional antibodies: Purified anti-CTLA-4 (clone 9D9), mouse IgG2b isotype (clone MPC-11) and anti-TGFβ (clone 1D11.16.8) were obtained from BioXCell. Anti-B7x was generated in-house from hybridoma culture.<br>Flow cytometry antibodies: The following fluorophore-conjugated antibodies were used for cell surface staining of mouse cells: anti-CD45 (BD), anti-CD3 (Biolegend), anti-CD4 (Biolegend), anti-CD8a (BD), anti-CD25 (Biolegend), anti-CD11b (Biolegend), anti-CD11c (Biolegend), anti- Ly6C (Biolegend), anti-Ly6G (Biolegend), anti-NK1.1 (Biolegend), anti-Neuropilin-1 (Biolegend), anti-F4/80 (Biolegend), anti-PD-1 (Biolegend), anti-Tim-3 (Tonbo), anti-TGF-LAP (Biolegend). Intracellular staining was performed with the eBioscience Transcription Factor Staining Buffer (Thermofisher) for the following antibodies: anti-Foxp3 (Thermofisher), anti-IFN-γ (Biolegend), anti-Helios (Biolegend). For phospho-flow cytometry of CD4+ T cells, cells were stained for: anti-p-Smad2/3 (BD), anti-p-p65 (CST), anti-p-c-Jun (CST), anti-p-Akt (CST), anti-p-Foxo1 (CST), anti-p-STAT1 (CST), anti-p-STAT3 (Biolegend), anti-p-STAT4 (BD), or anti-p-STAT5 (BD). Unconjugated antibodies were detected with anti-Rabbit Alexa 647 secondary antibody (Thermofisher).<br>Please refer to Supplementary Table 1 for clone numbers, catalog numbers, dilutions, and conjugated fluorophores of flow cytometry antibodies.
Validation	The antibodies used for flow cytometry in this manuscript are commonly used, commercially available clones. In particular, the primary antibody vendors used in this project (Biolegend and BD) carry out extensive validation processes for flow cytometry antibodies. These are selected from high-affinity clones, tested for binding across multiple assays, and/or specific binding confirmed by gene knockout of the target protein.<br>In this paper, expression of key markers including Foxp3, CD25, CTLA-4, B7x, TGF-LAP, Helios, Nrp1 was compared against Isotype

staining or fluorescence minus-one controls, and no non-specific staining was seen.

Eukaryotic cell lines

Policy information about cell lines

Cell line source(s)	The MC38 and CT26 cell lines were obtained from the Einstein Cytogenetics Facility cell line repository. The SKBR-3, MDA MB-468, OVCAR-3, OVCAR-4, and NCI HC322M cell lines were derived from the National Cancer Institute Developmental Therapeutics Program cell line repository. The Hepa1-6 cell line was obtained from the Marion Bessin Liver Center at the Albert Einstein College of Medicine.
Authentication	Cell lines were authenticated by the suppliers and further confirmed in the lab by FACS.
Mycoplasma contamination	The cell lines were routinely tested for mycoplasma contamination and confirmed to be negative. Cell lines were treated with prophylactic Plasmocin to prevent contamination prior to creating long-term storage stocks.
Commonly misidentified lines (See ICLAC register)	No commonly misidentified lines were used

Animals and other organisms

Policy information about studies involving animals; ARRIVE guidelines recommended for reporting animal research

Laboratory animals	8-12 week old, female, wild type C57BL/6 (B6) and BALB/c mice were purchased from Charles River (Frederick, MD), as needed for tumor experiments. B6 Foxp3-GFP-DTR and B6 CD45.1 mouse strains were obtained from Jackson Laboratories (Bar Harbor, ME) and bred in-house.<br>The animals were housed in a Specific Pathogen Free facility, in 12 hour light/12 hour dark cycles with temperatures maintained between 65F to 75F.
Wild animals	Wild animals were not used
Field-collected samples	Field-collected samples were not used
Ethics oversight	All animal protocols were reviewed and approved by the Institutional Animal Care and Use Committee of the Albert Einstein College of Medicine.

Note that full information on the approval of the study protocol must also be provided in the manuscript.

Flow Cytometry

Plots

Confirm that:

- [x] The axis labels state the marker and fluorochrome used (e.g. CD4-FITC).
- [x] The axis scales are clearly visible. Include numbers along axes only for bottom left plot of group (a 'group' is an analysis of identical markers).
- [x] All plots are contour plots with outliers or pseudocolor plots.
- [x] A numerical value for number of cells or percentage (with statistics) is provided.

Methodology

Sample preparation	Tumors were chopped and incubated for 40 minutes, shaken continuously at 37°C, in dissociation cocktail: 200 IU/mL Collagenase IV (Gibco), 0.5 IU/mL Dispase I, and 100 U/mL DNase I in serum-free medium. Dissociated cell suspensions were strained through 100 µm cell strainers, and subsequently centrifuged on a discontinuous 40%-80% Percoll gradient. The resulting interphase was extracted and used for downstream experiments.
Instrument	Stained samples were analyzed on the BD LSR-II or BD FACSCalibur
Software	BD FACS Diva 8.0 was used for acquisition, Flowjo 10.7 was used for data analysis
Cell population abundance	FMO controls were used to determine appropriate positively-stained populations. In general, a stopping gate of 10,000 to 50,000 was used on total live CD45+ or CD3+ cells. Final gated populations generally ranged in 1000 to 10,000 cells, and samples with less than 50 events in the final gated population were excluded.
Gating strategy	See Supplementary Data Fig. 5 for gating strategies used for in vivo immunophenotyping

- [x] Tick this box to confirm that a figure exemplifying the gating strategy is provided in the Supplementary Information.
